# Study protocol: effects of the THAO-child health intervention program on the prevention of childhood obesity - The POIBC study

**DOI:** 10.1186/1471-2431-14-215

**Published:** 2014-08-29

**Authors:** Santiago F Gomez, Rafael Casas, Vanessa Taylor Palomo, Anna Martin Pujol, Montserrat Fíto, Helmut Schröder

**Affiliations:** 1 Fundación THAO, Barcelona, Spain; 2 Cardiovascular Risk and Nutrition Research Group (CARIN), IMIM (Hospital del Mar Medical Research Institute), Barcelona, Spain; 3 Current affilation: Asociación Española Contra el Cáncer, Barcelona, Spain; 4 CIBER Physiopathology of Obesity and Nutrition (CIBEROBN), Instituto de Salud Carlos III, Madrid, Spain; 5 CIBER Epidemiology and Public Health (CIBERESP), Instituto de Salud Carlos III, Madrid, Spain

**Keywords:** Obesity, Community based intervention program, Lifestyle

## Abstract

**Background:**

The speeding increase and the high prevalence of childhood obesity is a serious problem for Public Health. Community Based Interventions has been developed to combat against the childhood obesity epidemic. However little is known on the efficacy of these programs. Therefore, there is an urgent need to determine the effect of community based intervention on changes in lifestyle and surrogate measures of adiposity.

**Methods/design:**

Parallel intervention study including two thousand 2249 children aged 8 to 10 years ( 4^th^ and 5^th^ grade of elementary school) from 4 Spanish towns. The THAO-Child Health Program, a community based intervention, were implemented in 2 towns. Body weight, height, and waist circumferences were measured. Children recorded their dietary intake on a computer-based 24h recall. All children also completed validated computer based questionnaires to estimate physical activity, diet quality, eating behaviors, and quality of life and sleep. Additionally, parental diet quality and physical activity were assessed by validated questionnaires.

**Discussion:**

This study will provide insight in the efficacy of the THAO-Child Health Program to promote a healthy lifestyle. Additionally it will evaluate if lifestyle changes are accompanied by favorable weight management.

**Trial registration:**

Trial Registration Number ISRCTN68403446

## Background

Obesity can reverse the growing trend of life expectancy [1]. Adiposity is associated with an adverse cardiometabolic profile cardiovascular not only in adults but also in children [2]. In this context it is important to note that there is a high likelihood of tracking childhood obesity into adulthood [3].

The speeding growth of childhood overweight and obesity [4] is a serious problem for public health worldwide [5]. Spain has one of the highest prevalence rates of childhood overweight and obesity among OECD countries. A recent study reported prevalence rates of 25,3% and 9,6% of overweight and obesity, respectively, in Spanish children aged 8-13 years [6]. Childhood obesity has a multifactorial aetiology. Unhealthy lifestyle such as inadequate diet and low physical activity is strongly related with weight gain [7].

Community Based Interventions programs (CBI) are a holistic approach to prevent childhood obesity. CBI act from all key sectors that influence childhood development (family, school, health professionals, sports, media, shops and market). There is limit information on the efficacy of CBI in Europe. Results from the Fleurbaix Laventie Ville Santé (FLVS) study showed that the implementation of a CBI program resulted in less weight gain in the intervention towns compared with the control towns [8]. Based on these results the EPODE program started in 2004 in France.

The THAO-Child health program (TCHP) a community based intervention program is based on the EPODE methodology. The main objective of this program is to prevent childhood obesity by promoting healthy lifestyle among children and their families. The TCHP is implemented by municipalities with the political involvement of the Mayor-Councillors and other local project managers appointed by the Mayor or Councillors. The local project manager integrates all the key stakeholders involved actively in community life. The TCHP is currently implemented in 75 municipalities of 8 Spanish autonomous communities.

This manuscript describes the rational and design of the POIBC study aiming to determine the efficacy of the TCHP.

## Methods

### Study design

A parallel intervention study to determine the effect of the THAO-Child Health Program on weight management, physical activity, quality of life, diet and sleep quality, and habits and behaviours.

### Subjects

Two thousand two hundred forty nine children aged 8 to 10 years (4^th^ and 5^th^ grade of elementary school) were recruited from 4 Catalan cities (Terrassa, Sant Boi de Llobregat, Molins de Rei, and Gavà) from September to November 2012. The Thao-Child Health Program was implemented in Terrassa y Sant Boi de Llobregat whereas Molins de Rei and Gavà serve as control cities.

### Sample size calculation

The sample size calculation is based on data on 1 year changes in BMI of children from 14 towns where the THAO intervention program has been implemented. A decrease in BMI of 0.55 kg/m^2^ was observed. Therefore, we considered reasonable to assume a difference in BMI of 0,6 kg/m^2^ between control and intervention group. 1070 participants in each group are necessary to detect a difference in BMI of 0.6 kg/m^2^ or higher assuming a 0,05 alpha risk and 0,2 beta risk in a bilateral contrast. A BMI standard deviation of 4.43 kg/m^2^ was assumed and 20% of missing during follow up was estimated.

### Intervention

The THAO-Child Health Program is being implemented in Terrassa and Sant Boi de Llobregat, with Molins de Rei and Gavà acting as control cities. Repeated measures of dietary intake and behaviour, physical activity, sleep quality, quality of life, and anthropometric variables will be performed during 2 years among all participating children. Additionally, parental sociodemographic variables, diet quality, and physical activity will be recorded. Computer-based software and questionnaires have been developed to record children’s lifestyle habits.

**Figure 1:**
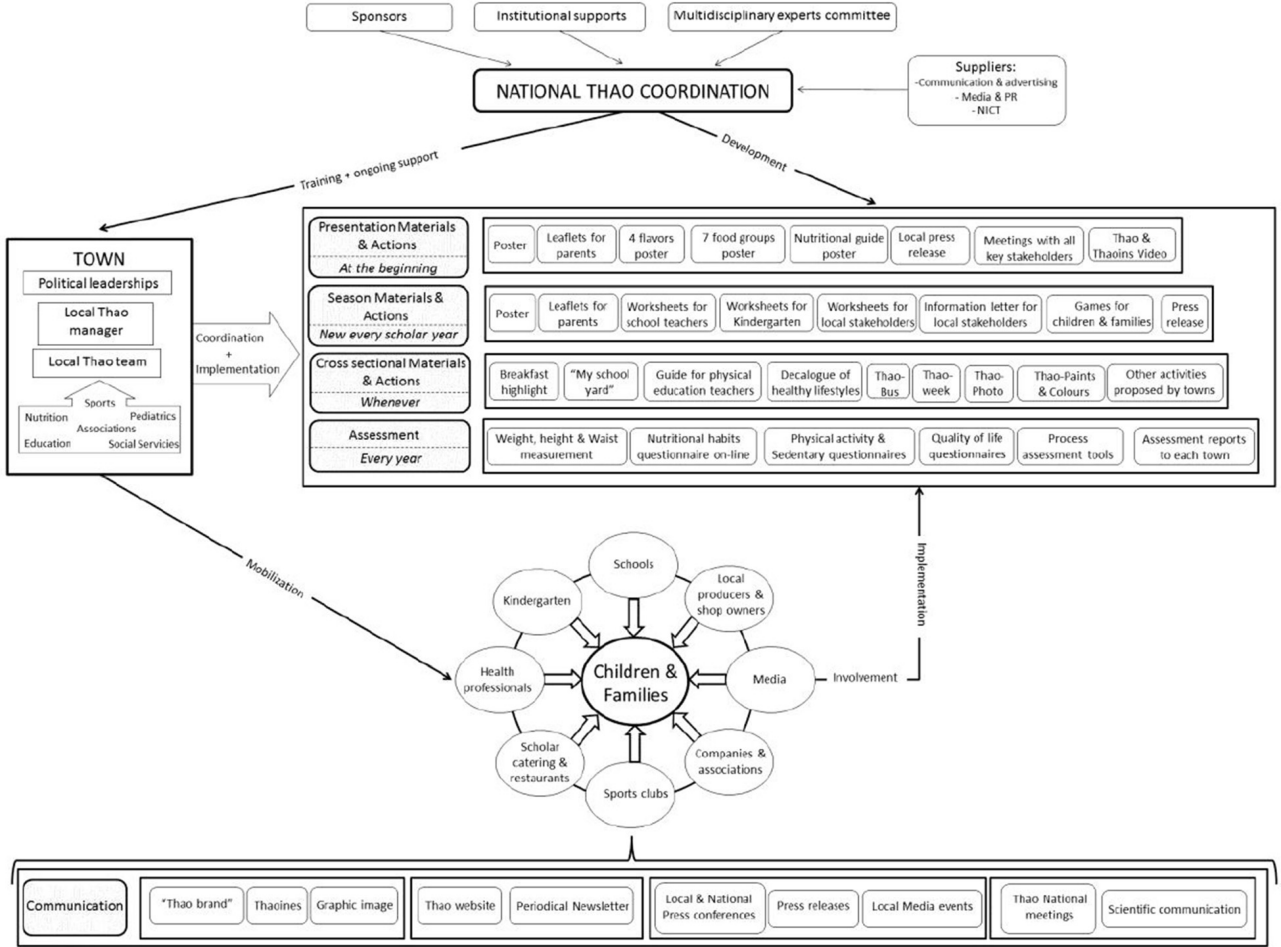
THAO-Child health program implementation methodology in towns.

The main objective of the THAO-Child Health Program is the prevention of childhood obesity through the promotion of healthy lifestyles of children and their families. THAO-Child Health Program it’s leaded by the city council which appoints the local coordinator who is supported by a multidisciplinary local team to reach all key sectors (family, school, health professionals, sports, media, shops and market). It’s a complete multisetting and multistrategy CBI.The Thao Foundation coordinate networks which develop the public health strategy, create the graphic materials and activities to all local key sectors. Furthermore, it gives the initial and the periodical training to local teams and coordinators and provides a constant support and annual evaluation to each town involved. All actions are being communicated by multiple channels (Figure 1).

### Data collection in children

#### Anthropometric variables

Anthropometric measurements are assessed for each individual following standard protocol by trained personnel. Body weight, height, and waist circumferences were measured on the same day of the first interview with the subjects wearing a t-shirt and light trousers. The measurements are performed without shoes and using an electronic scale (SECA 813), to the nearest of 100 g, a portable SECA 213 stadiometer (to the nearest 1 mm), and a metric tape (to the nearest 1 mm). Using a flexible non-stretch tape measure, waist circumference was measured by trained interviewers in the narrowest zone between the lower costal rib and iliac crest, in the supine decubitus and horizontal positions. Measuring devices are systematically calibrated.

#### Online software

All lifestyle data are self-reported with the assistance of trained personnel. Dietary intake information based is collected by a new on-line program at each participant’s school. This program consists of a single and structured 24-h recall. Photographs were provided for all foods and beverages. Additionally, photographs of different portion sizes of most consumed foods of Spanish children aged 8 to 10 years are available.

The online program includes, beside the 24h recall, the following questionnaires

1. The KIDMED questionnaire [9]

2. The Dutch Eating Behaviour Questionnaire for Children (DEBQ-C) [10]

3. An eating habit questionnaire.

4. The Physical Activity Questionnaire for Children (PAQ-C) [11].

5. The KIDSCREEN-10 questionnaire [12]

#### The KIDMED questionnaire

The KIDMED index was derived on the basis of a 16-item questionnaire administered separately from the 24-hour recalls as part of the enKID survey [9]. KIDMED was created to estimate adherence to the Mediterranean diet in children and young adults, based on the principles that sustain Mediterranean dietary patterns and those that undermine it. Items denoting lower adherence were assigned a value of -1 (4 items) and those related to higher adherence were scored +1 (12 items). Scores range from -4 to 12, with higher scores indicating greater adherence to the Mediterranean diet. A low adherence to the Mediterranean diet was defined as scoring below 6 points for the KIDMED index.

#### Eating behaviours

Eating behaviours were determined by the validated Dutch Eating Behaviour Questionnaire for Children (DEBQ-C) for use with Spanish children [10]. The DEBQ-C is a questionnaire adapted to age (7 to 12 years old), which assesses the presence of *External Eating*, *Emotional Eating*, and *Restrained Eating*. It is a self-applied questionnaire composed of 20 Likert type questions.

#### Physical activity assessment

Level of physical activity (PA) is assessed by the Physical Activity Questionnaire for Children (PAQ-C). The PAQ-C asks about different activities to define the PA level of the last week (the last 7 days) [11]. It provides a summary PA score derived from nine items. Each question is scored on a 5-point scale, with higher scores indicating higher levels of activity. The first question is a checklist of 22 common leisure and sports activities. The PAQ-C is widely accepted [13-15] and recommended [16] for international and national studies. In addition, a systematic review of measurement properties of self-report PA questionnaires for children concluded good to moderate validity and reliability of the PAQ-C [15]. The Physical Activity Questionnaire for Older Children (PAQ-C) and for Adolescents (PAQ-A) are self-administered, 7-day recall instruments, which were designed to provide a general estimate of PA levels in 8–20-year-old youth during the school year. Questionnaire items include weekly participation in different types of activities and sports (activity checklist), effort during physical education (PE), and activity during lunch, after school, evening and at the weekend.

#### Quality of life

Quality of life is assessed by the KIDSCREEN-10 questionnaire [12]. The KIDSCREEN-10 is a valid measurement tool of health related quality of life in children and adolescents. This questionnaire is the shortest version of three questionnaires (KIDSCREEN-52, KIDSCREEN-27, and KIDSCREEN-10). The KIDSCREEN-10 score contains 10 items. Each item is answered on a 5-point response scale. The KIDSCREEN- 10 Item statements are: (1) Have you felt fit and well? (2) Have you felt full of energy? (3) Have you felt sad? (4) Have you felt lonely? (5) Have you had enough time for yourself? (6) Have you been able to do the things that you want to do in your free time? (7) Have your parent(s) treated you fairly? (8) Have you had fun with your friends? (9) Have you got on well at school? (10) Have you been able to pay attention?

#### Sleep duration and quality

Parents answered to the questions of the Spanish version of the Pediatric sleep questionnaire (PSQ) to report on children’s sleep duration and quality [17]. The PSQ is a reliable measure for assessing SRBP in children, and has demonstrated valid results in a pediatric population compared with polysomnography (PSG) [18] The PSQ consists of 22 items and three subscales that examine snoring, daytime sleepiness, and daytime behavior. The PSQ scores are averaged so values range from 0 to 1 and are assessed as a continuous variable [18]. Parents were also asked to report the usual earliest and latest time their child went to bed and woke up for weekdays and weekends separately. Sleep duration was calculated as the number of hours on weekdays between the average of the usual earliest and latest bed time and the average of the earliest and latest times the child woke up.

### Data collection in parents

#### Dietary assessment

Diet quality is recorded by the short Diet Quality Screener (sDQS) [19]. Parents are asked to base their responses on their usual dietary behaviors over the previous 12 months, reporting their habitual intake of 18 food items grouped in 3 food categories.

#### Physical activity

The short version of the Minnesota Leisure-Time physical Activity Questionnaire (sMLTPA) is administered to estimate parents time spend in leisure physical activities. The sMLTPA consists of 5 questions on leisure physical activities that explain about 90% variability of all activities of the long version of the MLTPA.

#### Socioeconomic status

Is recorded by a standard questionnaire.

### Evaluation plan

The evaluation plan will be carried out during two scholar years. All variables will be collected in parents and children at the beginning of the 1^st^ scholar year and at the end of the 2^nd^ scholar year. An intermediate data collection at the beginning of the 2^nd^ scholar will be performed in children.

### Statistic

Data clean-up will be performed to minimize errors. Linear multivariate mixed models will be fit to analyze differences in changes in quantitative variables between groups. Pre-post changes will be considered as the response variable, and participants’ (age, gender, etc.) and municipalities’ characteristics (group membership) will be included as fixed effects explanatory variables. Additionally, to account for the hierarchical structure of the data, municipalities (Terrassa, Sant Boi de Llobregat, Molins de Rei, and Gavà) will be added to the model as random effects factors.

### Ethical issues

Parent consent was requested for each children and were performed parent meetings as requested by the schools. At any time the children or their family can leave the study and the data is automatically deleted.

The collection of anthropometric variables was performed in strict privacy conditions and gender dependent.

The study protocol was approved by the ethical committee of IMAS – Parc de Salut Mar, Barcelona, Spain.

## Discussion

The obesity epidemic is one of the biggest current challenges for health policy. The economic burden of obesity is estimated to be at around 10 percent of total health care costs [20]. Obesity prevalence has reached epidemic proportions and is associated with numerous cardiovascular risk factors in adults and children [2]. About 60% of the Spanish adult population are overweight or obese [21]. But most alarming is the high proportion of Spanish children and adolescents with excessive body weight. Currently four out of ten Spanish children between the ages of 8 and 17 suffer from excessive body weight [6]. This reflects one of the highest prevalence rates of childhood obesity among European countries [22]. This is of particular concern given the high probability that childhood obesity tends to continue into adulthood.

Therefore, intervention programs to prevent childhood obesity are needed. This in turn will improve the health status of children and reduce the economic burden of obesity. Several CBI programs that include families and key local community members have been developed mainly in the United States [23]. Some [24-27] but not all [28-32] of these CBI programs showed a favorable impact on lifestyle and anthropometric surrogate measures of adiposity.

A significant reduction in BMI z-scores has been reported by four CBI programs [24-27]. Additionally, de Silva-Sanigorski and colleagues showed an improvement in diet quality [26]. Improved physical fitness and an increase in physical activity were reported by Chomitz et al. [25] and Sallis et al. [24], respectively.

There is little data about the effect of CBI programs in European countries [28,29]. Therefore, it is paramount to develop and implement CBI programs tailored for country specific conditions in Europe. The POIBC study aims to determine the efficacy of the THAO-Child Health Program, a multisetting and multistrategy CBI program based on EPODE methodology [33]. The THAO-Child Health Program has been implemented in 75 towns in Spain since 2007. A basic characteristic of this program is the coordination by local authorities (municipalities) and the involvement of key sectors, such as schools, kindergarten, markets, sports and leisure time associations, health care providers and other local institutions relevant for child health. The TCHP includes health promotion materials and actions, permanent training of local coordinators and the development of an assessment and communication plan. This study represents an advance with respect to previously reported interventions in childhood obesity because the TCHP represents a sustainable intervention for municipalities and offers the possibility of maintain existing actions. Furthermore, the actions are carried out under the same communication model, and cartoon characters called “Thaoines” facilitate the health education of children. The TCHP is based on the I-Change Model defined by de Vries (last updated I-Change Model 2.0, 2008 [34]). This model is an amplification of the ASE model [Attitude – Social influence – self Efficacy Model [35]). The basic concept of the I-Change Model is an integration of ideas from Ajzen’s Theory of Planned Behaviour] [36], Bandura’s Social Cognitive Theory [37], Prochaska’s Transtheoretical Model [38], the Health Belief Model [39] and goal setting theories [40].

The POIBC study includes 2 intervention and 2 control towns and monitorate anthropometric variables and obesity related behaviour. Furthermore, parental socioeconomic status and lifestyle are recorded. An innovative aspect of the POIC study is the implementation of an on-line questionnaires to record lifestyle variables in children. Compared with the paper versions, the on-line software allows the children to answer in a more dynamic way. This, in turn, may help reduce mistakes in the response process.

A limitation of the POIBC study is its non-representative design. However, intervention and control towns have similar sociodemographic and socioeconomic characteristics, and are located in the same geographical area.

The POIBC study assesses changes in intervention and control towns during two complete academic years. This study will provide evidence about the efficacy of THAO-Child Health Program in Spain to confront the childhood obesity epidemic. Furthermore, it will give us a better understanding of the impact of this program on the development of obesity related behaviours.

## Abbreviations

CBI: Community based interventions programs; sDQS: Diet quality screener; DEBQ-C: Dutch eating behaviour questionnaire for children; EPODE: Ensemble Prévenons l'ObésitéDes Enfants; OECD: Organisation for Economic Co-operation and Development; PSQ: Pediatric sleep questionnaire; PAQ-C: Physical Activity Questionnaire for Children (PAQ-C); sMLTPA: Short version of the Minnesota leisure-time physical activity questionnaire; TCHP: THAO-Child health program.

## Competing interests

The authors declare that they have no competing interests.

## Authors’ contributions

SFG, RC, and HS were responsible for the study concept, design, and funding. All authors contributed to the protocol design. SFG and HS led the development of the online Software. SFG, VTP, AMP and MF oversaw participant recruitment and intervention. SFG led the drafting of this manuscript, with input from all authors. All authors have read and approved the final version of the manuscript.

## Pre-publication history

The pre-publication history for this paper can be accessed here:

http://www.biomedcentral.com/1471-2431/14/215/prepub
